# Integrated mosquito larval source management reduces larval numbers in two highland villages in western Kenya

**DOI:** 10.1186/1471-2458-12-362

**Published:** 2012-05-18

**Authors:** Susan S Imbahale, Andrew Githeko, Wolfgang R Mukabana, Willem Takken

**Affiliations:** 1 Laboratory of Entomology, Wageningen University, P.O. Box 8031, 6700 EH, Wageningen, The Netherlands; 2 Kenya Medical Research Institute, Centre for Global Health Research, P.O Box 1578-40100, Kisumu, Kenya; 3 International Centre of Insect Physiology and Ecology, P.O. Box 30772 – 00100 GPO, Nairobi, Kenya; 4 School of Biological Sciences, University of Nairobi, P.O. Box 30197-00100 GPO, Nairobi, Kenya; 5 School of Pure and Applied Sciences, Kenya Polytechnic University College, P.O Box 52428-00200, Nairobi, Kenya

**Keywords:** *Anopheles* spp, Larval source management, *Bacillus thuringiensis* var *israelensis* (*Bti*), Drainage, *Gambusia affinis*, Arrow root, Kenya

## Abstract

**Background:**

In western Kenya, malaria remains one of the major health problems and its control remains an important public health measure. Malaria control is by either use of drugs to treat patients infected with malaria parasites or by controlling the vectors. Vector control may target the free living adult or aquatic (larval) stages of mosquito. The most commonly applied control strategies target indoor resting mosquitoes. However, because mosquitoes spend a considerable time in water, targeting the aquatic stages can complement well with existing adult control measures.

**Methods:**

Larval source management (LSM) of malaria vectors was examined in two villages i.e. Fort Ternan and Lunyerere, with the aim of testing strategies that can easily be accessed by the affected communities. Intervention strategies applied include environmental management through source reduction (drainage of canals, land levelling or by filling ditches with soil), habitat manipulation (by provision of shading from arrow root plant), application of *Bacillus thuringiensis* var *israelensis* (*Bti*) and the use of predatory fish, *Gambusia affinis*. The abundance of immature stages of *Anopheles* and Culex within intervention habitats was compared to that within non-intervention habitats.

**Results:**

The findings show that in Fort Ternan no significant differences were observed in the abundance of *Anopheles* early and late instars between intervention and non-intervention habitats. In Lunyerere, the abundance of *Anopheles* early instars was fifty five times more likely to be present within non-intervention habitats than in habitats under drainage. No differences in early instars abundance were observed between non-intervention and habitats applied with *Bti.* However, late instars had 89 % and 91 % chance of being sampled from non-intervention rather than habitats under drainage and those applied with *Bti* respectively.

**Conclusion:**

Most of these interventions were applied in habitats that arose due to human activities. Involvement of community members in control programs would be beneficial in the long term once they understand the role they play in malaria transmission. Apart from the need for communities to be educated on their role in malaria transmission, there is a need to develop and test strategies that can easily be accessed and hence be used by the affected communities. The proposed LSM strategies target outdoor immature mosquitoes and hence can complement well with control measures that target indoor resting vectors. Therefore inclusion of LSM in Integrated Vector Management (IVM) program would be beneficial.

## Background

Malaria is endemic in many regions of East Africa where climate and environment together present conditions suitable for malaria vectors and parasites [1]. The main vector species in western Kenya are *Anopheles gambiae* Giles *sensu strictoAn. arabiensis* Patton and *An. funestus* Giles. *Anopheles gambiae* and *An. arabiensis* are commonly found in clear sunlit pools of water, man-made shallow water bodies, in polluted water and along the shores of large water bodies such as Lake Victoria [2-8]. *Anopheles funestus* prefers rather permanent water bodies (8). In the last decade an increasing number of cases of malaria in formerly malaria-free areas and highland areas have become common [9-11]. Several hypotheses have been proposed to explain the increased malaria transmission in the highlands, including land-use changes, global climate changes, increased drug resistance, cessation of malaria control activities, and demographic changes [11-13]. Cox [14] estimated that 34 million individuals were at risk of malaria in the East African highlands. In these highlands, transmission is probably much more focal in its distribution than in many lowland areas, as breeding sites are more common in the valley floor than on the steep valley slopes [15]. In addition, studies report that human activities in these highlands have subsequently created potential mosquito breeding habitats [7,11,16].

In the Ugandan highlands, the elimination of papyrus swamps created a habitat for *An. gambiae* and *An. funestus*, leading to increased malaria transmission [11]. In the highlands of western Kenya, *An. gambiae* was found only in cultivated farmland habitats but not in original forest and swamp habitats [16]. These differences in larval distribution were attributed to the fact that farmland habitats received more sunlight, and hence water temperatures were conducive for *An. gambiae* breeding. In Ethiopia, changes in land use and climate expose the highland areas to unexpected malaria epidemics, presumably due to expansion of environmental conditions suitable for malaria transmission [17].

Research on malaria in the highlands has mainly focused on the development of early-warning systems to identify when epidemics are expected [18-20] and on the effects of changes in climatic variables [13,21,22]. The core idea behind these systems is that when parameters indicate a malaria epidemic is likely, resources can be channeled to prevent or contain the epidemic [18]. However, in sub-Saharan Africa, malaria epidemics arise suddenly in mostly remote, disadvantaged settings without effective alert systems [23]. In resource-limited countries such as those of highland East Africa, an all-or nothing approach to interventions such as insecticide spraying or bed net distribution, often results in complete coverage for some areas and no coverage for others when funds run out [18]. Thus, regular vector control activities targeted at the malaria risk areas are more cost effective than emergency interventions that often face delays in mobilization [24]. In addition, because full coverage of control measures is hardly achieved, integration of larval source management (LSM) into Integrated Vector Management (IVM) program will be advantageous to the fight against malaria.

In western Kenya highlands, for instance, since the implementation of the roll back malaria initiative [25], malaria control has been based on insecticide treated nets (ITNs), indoor residual spraying (IRS) and the use of anti-malarial drugs for the treatment of malaria parasites. Following the adoption of RBM, there are indications that malaria morbidity and mortality is on a decline as a result of scaled up use of ITNs [26,27] and increased availability of antimalarial medicines [28]. However, with increased use of interventions targeting indoor resting mosquitoes, the vectors are bound to develop evading mechanisms or even change their biting behavior. Exophily of the commonly known endophilic species has recently been reported [29,30], in addition to development of resistance in the malaria vector and parasites. There is need development and integration of complementary tools to target outdoor vectors [31,32].

Microbial larvicides have been proven efficient in the control of anopheline mosquito larvae and the reduction in adult mosquito densities [33-36]. However, access to microbial larvicides is still a challenge for developing countries, thus calling for development of alternative larval control strategies that can utilize locally available resources. In the current study, an integrated larval source management comprising of habitat manipulation, source reduction in comparison to the application of microbial larvicides and the use predatory fish were used. The hypothesis being habitat manipulation and source reduction are as effective as the application of *Bti* and the use of predatory fish for mosquito larval control.

## Methods

### Study area

The study was implemented in two rural highland villages, Lunyerere (0°06’North and 34°43’East) in Vihiga District at 1520 to 1560 m and Fort Ternan (0°12’South and 35°20’East) in Kericho District at 1500 to 1650 m above sea level both from western Kenya. Western Kenya has a bimodal pattern of rainfall with long rains occurring from April to June and short rains between November and December with yearly variations. The study area has been described in Imbahale and others [7]. Briefly, Lunyerere is situated between undulating hills whose foot are large basin shaped valleys in which surface runoff water collects resulting in extended swamps. Houses in Lunyerere are spread over a large area. In contrast, Fort Ternan is located on the slopes of Nandi hills, an area that is characterized by steep slopes forming sharp V-shaped valleys providing less room for standing water. Baseline entomological data (adult and larval mosquito densities) collected between March 2006 to March 2008 showed that *An. gambiae* s.l*,* is the principal malaria vector in both villages [7]. *Anopheles gambiae* was found to occupy temporary habitats (e.g. water pools) more often in Fort Ternan, whereas in Lunyerere both temporary and permanent habitats (mainly drainage canals) were equally inhabited. For this reason, interventions targeting mosquito larvae were applied to both temporary and permanent habitats. However, because of the transient nature of temporary larval breeding habitats, larviciding was the most suitable strategy used. For permanent habitats, the selection relied mainly on the suitability of the habitat for a given intervention for example source reduction was more suited for drainage canals, whereas predatory fish was best for pond-like habitats.

### Larval source management strategies

#### Larviciding

Water-dispersible and granulated formulations of the commercial larvicide VectoBac® containing *Bacillus thuringiensis* var. *israelensis* (*Bti*; Valent Biosciences Corporation, Libertyville, IL, USA) was applied to all temporary habitats and selected permanent habitats in Fort Ternan and Lunyerere. The microbial larvicide was broadcasted on the larval habitats at weekly intervals at an optimum dosage and concentration of 200 g/ha [37].

#### Predatory fish

A colony of *Gambusia affinis* (Cyprinodontiformes: Poeciliidae) was initiated from wild-caught samples with the help of staff from Kenya Marine and Fisheries Research Institute (KEMFRI) resident in Kisumu, Kenya. The fish colony was maintained at Fort Ternan, in man-made ponds under natural conditions. The fish population was left to establish from June to July 2008, while being fed on fish food supplement provided by KEMFRI. In August, introductions of mosquito fish were made into pond-like habitats, based on a laboratory-determined ratio of four fish per 60 larvae [38]. Mosquito fish were introduced into the respective habitats only once and the population left to increase naturally. No supplementary food was provided after fish was introduced into habitats.

#### Source reduction

Source reduction was achieved through drainage of canals, land levelling or by filling ditches with soil. In Fort Ternan, water was drained off by a natural gradient into a main canal, which flowed into a Kipchorian river. Habitats subjected to drainage were checked weekly to remove any unwanted debris that could reduce or stop water movement. Habitats located along the river fringes were filled and levelled using stones and/or soil to prevent any water from stagnating. In areas where water stagnated due to debris in the river, the debris was removed to allow for easy flow of water.

#### Habitat manipulation

This refers to activities that reduce vector larval breeding habitats through temporary changes in the aquatic environment where larvae develop [39]. Breeding habitats were shaded with arrow root (*Maranta arudinacea*) crops planted along selected water canals in Lunyerere to provide temporary non-conducive conditions (reduced temperatures) for mosquito breeding. Arrow root plants were selected for this study as they are locally available as a source of food and are mainly grown in swampy areas. The seedlings were locally obtained from resident farmers. Previous field trials showed that mosquito breeding habitats shaded by arrow root plants had significantly reduced immature anopheline mosquito populations [38].

### Implementation programme of LSM strategies

#### Fort Ternan

This study was conducted for a period of eight months, from August 2008 to March 2009 and rains were experienced from September to October 2008 and from April to June 2009. Productive larval habitats in Fort Ternan were rain pools, erosion pits, watering points and habitats along the river fringe originating from animal hoof prints, which left depressions that filled with water forming stagnant pools. In addition, debris carried by water into the river settled along the river fringe blocking the flow of water which resulted to formation of stagnant water pools. Twelve permanent mosquito breeding habitats i.e. erosion pits, watering points, drainage canals, were identified from baseline data. Three different intervention types were applied i.e., larviciding, source reduction and introduction of predatory fish. Each intervention was replicated three times and a fourth series of habitats was left without any intervention (non-intervention). Interventions were implemented depending on the suitability of the habitat; for example erosion pits were provided with predatory fish, breeding habitats occasioned by leaking taps were treated with *Bti,* while habitats along the river fringes were drained to allow water to flow, filled with soil or levelled off.

#### Lunyerere

This study was conducted for a period of 12 months, from April 2008 to March 2009. During the study the rains were experienced from April to June 2008 and 2009 and from November and December 2008. The main source of larval habitats was upwelling from underground sources that provided small pools of stagnant water in drainage canals, thus creating good breeding grounds [7]. Twenty-four permanent breeding habitats were selected, these being drainage canals. Three different LSM strategies were applied i.e., source reduction, habitat manipulation and larviciding. Each LSM strategy was replicated six times and a fourth series of water canals were left untreated (non-intervention). Water in the drainage canals was maintained by upwelling or rainfall.

### Post-intervention

The LSM strategies ended in March 2009. From April through June 2009 larval sampling within habitats that were previously drained and applied with *Bti* was carried out to investigate the re-establishment of mosquito larval populations. Habitats containing predatory fish and those planted with arrow roots were left intact during the post-intervention period.

#### Larval abundance

The primary entomological outcome was mosquito larval abundance which served to evaluate the effectiveness of the LSM strategies applied. Larval sampling was done once a week i.e. always on the fourth day after application of *Bti,* using the standard dipping method with a 350 ml mosquito scoop [Bioquip, Gardena CA, USA] [40]. Up to 10 dips were taken from each habitat and the larvae collected separated into anophelines and culicines, counted and recorded. The larvae were recorded either as early instars (L1 and L2) or late instars (L3 and L4). Late instar anopheline larvae were immediately preserved in 90 % absolute ethanol and taken to the laboratory at the Kenya Medical Research Institute (KEMRI), Kisumu, for taxonomic identification [41].

### Data analysis

Generalized Linear Model, univariate analysis with a normal probability distribution was used in the calculation of odds ratio (OR) and 95 % Wald Confidence Intervals. Mosquito larval abundance in habitats provided with different interventions was compared to that of non-intervention habitats. Analysis of variance (ANOVA) was used to find out the differences in larval abundance within habitats three months before and after termination of interventions. All analysis was carried out using SPSS 16.0 for Microsoft Windows.

## Results

### Larval dynamics

#### Fort Ternan

**Figure 1:**
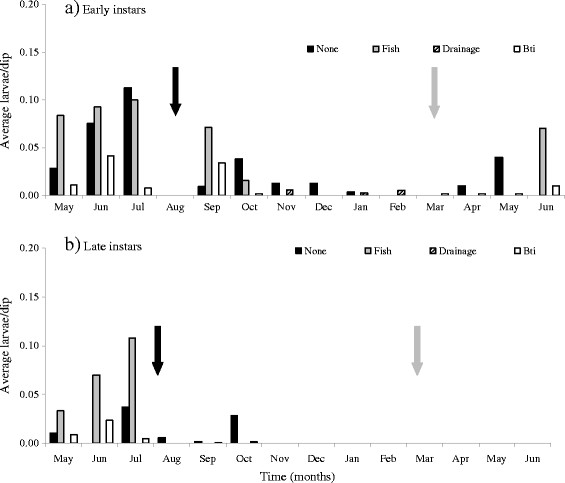
**Early (a) and late (b) instars monthly abundance of anopheline larvae in permanent habitats subjected to larval source management strategies in Fort Ternan from May 2008 to July 2009.** The black arrow indicates the start (August) and the grey the end (March) of larval source management measures.

The abundance of *Anopheles* early and late instars within intervention and the non-intervention habitats shows no significant (P > 0.05) differences (Table 1 and Figure 1). However, the abundance of early and late culicine instars was distributed differently within intervention and non-intervention habitats (Table 1). There was a 40 to 45 % (OR 0.544 - 0.591; P ≤ 0.001) likelihood of sampling culex early or late instars within the non-intervention rather than within habitats applied with Bti, fish or drainage.

**Table 1:** ***Anopheles*and Culex larval abundance within intervention and non-intervention habitats in Fort Ternan**

	**Weekly sampling (%)**	**Anopheles early (EM ± SE)**	**OR**	**P**	**Anopheles late (EM ± SE)**	**OR**	**P**	**Culex early (EM ± SE)**	**OR**	**P**	**Culex late (EM ± SE)**	**OR**	**P**
Non-intervention	25	0.18 ± 0.046	1		0.11 ± 0.037	1		0.83 ± 0.044	1		0.76 ± 0.050	1	
Bti	36	0.11 ± 0.031	0.931	> 0.05	0.02 ± 0.014	0.913	< 0.05	0.30 ± 0.045	0.587	< 0.05	0.16 ± 0.036	0.544	< 0.05
Fish	24	0.15 ± 0.044	0.969	> 0.05	0.00 ± 0.00	0.895	< 0.05	0.22 ± 0.051	0.544	< 0.05	0.16 ± 0.046	0.549	< 0.05
Drainage	15	0.09 ± 0.044	0.914	> 0.05	0.00 ± 0.00	0.895	< 0.05	0.31 ± 0.065	0.591	< 0.05	0.21 ± 0.057	0.576	< 0.05

#### Lunyerere

**Figure 2:**
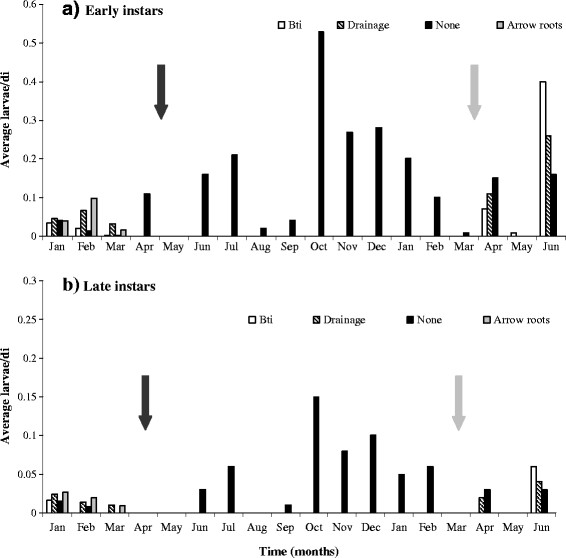
**Early (a) and late (b) instars monthly abundance of anopheline in permanent habitats subjected to LSM strategies in Lunyerere from Jan 2008 to July 2009.** The black arrow indicates the start (April) and the grey the end (March) of larval source management measures.

The abundance of *Anopheles* early instars was fifty five times (OR 0.45; P < 0.05) more likely to be sampled in non-intervention habitats than those under drainage (Table 2). Although no significant differences (OR 0.133; P = 0.05) were observed in the abundance of *Anopheles* late instars in drained and non-intervention habitats, there was 89 % chance of sampling late instars in non-intervention habitats rather than the drained habitats. On the other hand, the abundance of early instars within the non-intervention habitats and those applied with *Bti* was similar (OR 0.673; P >0.05). Contrary to this, the late instars were 91 % times more likely to be sampled within the non-intervention than within habitats applied with Bti. Early (OR 0.392; P <0.05) instars of Culex were more likely to be sampled in non-intervention habitats than in habitats applied with *Bti*, while the late instars abundance was marginally significant (OR 0.509; P =0.05). Due to lack of variance in the abundance of both *Anopheles* and Culex larvae in habitats provided with arrow roots, the odds ratio was not computed (Table 2). Figure 2 shows the monthly abundance of *Anopheles* larvae in habitats applied with different interventions.

**Table 2:** ***Anopheles*and Culex larval abundance within habitat provided with different interventions in Lunyerere**

	**Weekly sampling (%)**	**Anopheles early (EM ± SE)**	**OR**	**P**	**Anopheles late (EM ± SE)**	**OR**	**P**	**Culex early (EM ± SE)**	**OR**	**P**	**Culex late (EM ± SE)**	**OR**	**P**
Non-intervention	24	0.36 ± 0.030	1		0.32 ± 0.029	1		0.29 ± 0.028	1		0.29 ± 0.029	1	
Bti	23	0.00 ± 0.00	0.673	> 0.05	0.00 ± 0.00	0.088	< 0.05	0.04 ± 0.012	0.392	< 0.05	0.04 ± 0.012	0.509	< 0.05
Drainage	27	0.00 ± 0.00	0.451	< 0.05	0.00 ± 0.00	0.113	< 0.05	0.00 ± 0.00	0.661	> 0.05	0.00 ± 0.003	0.640	> 0.05
Yams	26	0.00 ± 0.00	1	a	0.00 ± 0.00	1	a	0.01 ± 0.005	1	a	0.01 ± 0.005	1	a

### Post-intervention period

Data for three months before (January to March 2009) and after (April – June 2009) termination of intervention strategies was used to find out any differences in *Anopheles* and Culex larval abundance within habitats that were subjected to different interventions. In Fort Ternan, *Anopheles* early and late instars were absent in non-intervention habitats and those provided with *Bti*, fish and drainage three months to end of intervention (Table 3). Minimal numbers of Culex early and late instars were sampled from non-intervention habitats. During the post intervention period no late instars of *Anopheles* were recorded from any of the habitats. However Culex early and late instars were present in non-intervention habitats and those previously applied with *Bti* and fish. Analysis of variance showed no significant differences in Anopheles early, culex early and late instars in intervention habitats while there was a lack of variance in Anopheles late instars abundance in the same habitats (Table 4).

**Table 3:** The mean larval abundance within habitats three months before and after termination of interventions within habitats in Fort Ternan and Lunyerere

**FORT TERNAN**								
	Last 3 months during intervention				3 months after intervention			
	Non-intervention	Bti	Fish	Drainage	Non-intervention	Bti	Fish	Drainage
Anopheles early	0.00 ± 0.002	0.00 ± 0.00	0.00 ± 0.00	0.00 ± 0.02	0.05 ± 0.012	0.00 ± 0.004	0.02 ± 0.015	0.00 ± 0.00
Anopheles late	0.00 ± 0.00	0.00 ± 0.00	0.00 ± 0.00	0.00 ± 0.00	0.00 ± 0.001	0.00 ± 0.00	0.00 ± 0.00	0.00 ± 0.00
Culex early	0.16 ± 0.05	0.00 ± 0.00	0.00 ± 0.005	0.01 ± 0.005	0.16 ± 0.020	0.18 ± 0.027	0.02 ± 0.012	0.00 ± 0.00
Culex late	0.13 ±0.029	0.003 ± 0.003	0.001 ± 0.001	0.004 ± 0.003	0.10 ± 0.011	0.10 ± 0.02	0.015 ± 0.0154	0.00 ± 0.00
**LUNYERERE**								
	Last 3 months during intervention				3 months after intervention			
	Non-intervention	Bti	Drainage	Arrow roots	Non-intervention	Bti	Drainage	Arrow roots
Anopheles early	0.19 ± 0.47	0.00 ± 0.00	0.00 ± 0.00	0.00 ± 0.00	0.09 ± 0.032	0.18 ± 0.045	0.13 ± 0.038	0.00 ± 0.00
Anopheles late	0.20 ± 0.049	0.00 ± 0.00	0.00 ± 0.00	0.00 ± 0.00	0.02 ± 0.005	0.02 ± 0.007	0.02 ± 0.008	0.00 ± 0.00
Culex early	0.25 ± 0.052	0.00 ± 0.00	0.00 ± 0.00	0.00 ± 0.00	0.15 ± 0.052	0.14 ± 0.046	0.26 ± 0.066	0.00 ± 0.00
Culex late	0.22 ± 0.050	0.00 ± 0.00	0.00 ± 0.00	0.00 ± 0.00	0.11 ± 0.043	0.16 ± 0.048	0.21 ± 0.053	0.00 ± 0.00

**Table 4:** ANOVA table giving results of a comparison of larval abundance before and after termination of interventions within habitats in Fort Ternan and Lunyerere

**Site**	**Intervention**	**Variable**	**Anopheles early**	**Anopheles late**	**Culex early**	**Culex late**
Fort Ternan	None	F	5.329	0.364	0.010	1.197
		P	< 0.05	> 0.05	> 0.05	> 0.05
	Bti	F	3.752	a	180.435	60.559
		P	= 0.055	a	< 0.05	< 0.05
	Fish	F	1.322	a	2.392	1.116
		P	> 0.05	a	> 0.05	> 0.05
	Drainage	F	0.271	a	0.767	0.241
		P	> 0.05	a	> 0.05	> 0.05
Lunyerere	None	F	2.870	13.334	1.887	2.676
		P	> 0.05	< 0.05	> 0.05	> 0.05
	Bti	F	14.436	11.066	8.384	8.867
		P	< 0.05	< 0.05	> 0.05	> 0.05
	Drainage	F	10.847	6.426	15.220	15.658
		P	< 0.05	< 0.05	< 0.05	< 0.05
	Arrow roots	F	a	a	a	a
		P	a	a	a	a

Note: a = no variance between the before and after group hence statistics was not computed.

P = 95 % significance level.

Degrees of freedom (df) =1 in all cases.

In Lunyerere 3 months before end of intervention larvae was only present in no-intervention habitats. However, once the interventions were stopped, habitats previously drained and those applied with *Bti* including the non-intervention habitats recorded both early and late stages of *Anopheles* and Culex larvae with the only exception being habitats provided with arrow roots (Table 3). Analysis of variance showed significant differences in abundance of larvae in intervention habitats provided with *Bti* and drainage whereas no variation was observed in habitats provided with shade from arrow roots (Table 4).

### Species composition

A total of 30 and 163 *Anopheles* late instar larvae were collected from the non-intervention habitats in Fort Ternan and Lunyerere, respectively. Among these, 7 % (2/30) from Fort Ternan and 9 % (14/163) from Lunyerere were *An. gambiae* sensu lato*.* Ninety three percent (28/30) of the anophelines in Fort Ternan were non-vector species mainly dominated by *An. marshalii*. *Anopheles funestus* (4/163) and *An. coustani* (2/163) were only found in Lunyerere while the remaining 87 % (143/163) comprised of non-vector anopheline species such as *An. marshalii* and *An. coustani*.

## Discussion

The results obtained indicate that the LSM intervention strategies used were effective in reducing the development of late instars larvae as few to none were recorded especially from intervention habitats. Non-intervention habitats on the other hand recorded both *Anopheles* and Culex larvae throughout the study in Lunyerere with the exception of Fort Ternan where no larvae was recorded during some of the months. An integrated LSM approach using environmental management through application of *Bti*, source reduction, habitat manipulation and the use of predatory fish showed great potential in preventing development of mosquito larvae in man-made habitats within the study area. Strategies such as the use of arrow root plants and drainage can be applied by the local communities for the control of mosquitoes in the respective study areas as they compare well with the use of predatory fish and the application of *Bti.* Culicine mosquitoes, mainly nuisance biters were also reduced in the intervention habitats.

Anopheline mosquito species breed in a variety of habitats; however, those created by human activities may be of particular importance for malaria transmission [6,7,39]. The bio-larvicide *(Bti)* although efficient in controlling mosquito larvae, it is not locally available and hence not accessible by the local community members. Therefore *Bti* was used alongside environmental management through habitat manipulation by use of shade provided by arrow roots and source reduction by drainage. The results show that in Lunyerere both early and late instar anopheline larvae were greatly reduced or absent in habitats shaded by the leaves of arrow roots plants and those that were drained. The abundance of *Anopheles* early instars within habitats treated with *Bti* and non-intervention was not different. Indicating that ovipositing females still lay their eggs in habitats containing *Bti* and as a result of feeding on *Bti* contained in water, the larvae then dies before reaching late instar increasing *Bti’*s efficiency in killing larvae. Results obtained from Fort Ternan show a similar pattern, where early instars were recorded from *Bti* intervention habitats while the late instars were fewer within habitats provided with *Bti*, predatory fish and those under drainage. The efficacy of *Bti* in reducing mosquito larval populations recorded in this study is comparable to the studies by Majambere et al. [36] and Fillinger et al. [37]. No residual effect of *Bti* was observed during the post intervention period as both early and late instar anopheline and Culex larvae were present within habitats previously applied with *Bti*.

The use of predatory fish for mosquito control has not been widely used in Africa; however, large scale trials using various species of larvivorous fish to control anopheline and culicine larvae have been reported in the Mediterranean region [42]. *Gambusia affinis* has been in use over a long period of time for mosquito control in countries such as Afghanistan, Cyprus, Egypt, Sudan and Jordan. Tilapia (*Oreochromis*) and *Aphanius dispar* are other species that are in use for mosquito control [42]. In western Kenya, Howard et al. [43] have shown the potential of *Oreochromis niloticus,* for mosquito control. In the current study when predatory fish*, G. affinis* was used as a single option in erosion pits, few early instars were recorded whereas no late instars were recorded from the same habitats. The results indicate that the female mosquitoes would still oviposit within habitats with fish but the larvae are fed upon before reaching their late stages.

In Lunyerere, manipulation of breeding habitats through shade provision by growing Napier grass [44] or arrow roots grown along drainage canals are capable and promising in the control of anopheline larvae. Source reduction involved modification of the existing canals to increase water flow so that larvae would be flushed out into a fast moving water canal/ river where they eventually die. No anopheline and culicine larvae were recorded from drained habitats in Lunyerere and Fort Ternan. If well implemented, drainage is successful; however the open drains or drainage canals need to be maintained to remove any debris that may slow water flow and lead to creation of pools of stagnant water that provide mosquito grounds. A number of studies have reported the successes of malaria reduction and eradication through environmental management projects [45-47]. However, such strategies have not been fully exploited in areas where malaria still remains a major health problem such as in western Kenya.

The LSM strategies were executed differently in the two study sites because of the variation in the nature of the breeding habitats. Lunyerere being a reclaimed swamp area, drainage canals were made for land reclamation to give way for farming. Failure of maintenance of the drainage canals led to creation of stagnant pools of water that are preferred mosquito breeding habitats. The findings show that source reduction through drainage and habitat manipulation compared well with larviciding. Arrow roots do well in swampy areas and because they are a good source of carbohydrates, they ensure food security for the land owner/farmer, in-addition, they may also be sold to generate income. On the other hand in Fort Ternan, stagnant water resulting from leakage of water pipes/taps and pools of water on the fringes of Kipchorian river were drained, whereas erosion pits formed pond like habitats that were stocked with predatory fish. The alternatives strategies applied in both sites compared well with the application of *Bti.* For a reclaimed swamp area environmental management would work well while in areas with pond-like habitats the use of predatory fish would be beneficial. However, a number of limitations were experienced during the study. The results for Fort Ternan should be interpreted carefully because in this area, breeding habitats were fewer when compared to Lunyerere, thus limiting the number of replicates. Previous studies in the same area found the densities of immature malaria vectors to be very low [7]. The sampling period coincided with the dry period (November to February) and a number of potential breeding habitats dried out further reducing the number of replicates. Thus Fort Ternan might not have been a good site for such as trial when compared to Lunyerere where the vector breeding was throughout due to the presence of favourable breeding grounds. Nevertheless, the results are promising and future studies should be done in an area where vectors breed in more habitats to allow for a better judgement of the strategies applied.

Recent times have witnessed the successes of an integrated approach of malaria control in many countries through the combination of ITNs and IRS as advocated by the Roll Back Malaria initiative [25]. Although insecticide treated bednets and indoor residual spray are highly effective for the control of indoor biting and resting mosquitoes, due to vector avoidance and possible behaviour changes [29,30,48] strategies targeting outdoor vectors are required to complement existing measures. In a previous study, it was found that the community members were willing to take part in larval source reduction but they lacked evidence-based results on control strategies that can be used with locally available resources [49]. As a step towards achieving this goal, the manuscript provides results of a small scale field trial on LSM strategies that can be incorporated into an IVM program, using locally available resources in comparison to the application of larvicides and the use of predatory fish for mosquito larval control.

## Conclusion

Most of the mosquito breeding habitats were man-made, resulting from land use changes associated with activities such as deforestation, swamp reclamation and animal husbandry [7]. Involvement of these communities in mosquito control interventions is expected to lead to more sustainable malaria control than is currently the case [50,51]. Larval Source Management strategies such as habitat manipulation and source reduction can be integrated in IVM programs and into the activities of the farming communities. Future studies should investigate to what extent the local community are willing to participate in these interventions.

## Competing interests

The authors declare that there is no conflict of interest.

## Authors’ contributions

This study was conceived by Imbahale SS and Takken W. Imbahale SS supervised field data collection, did data analysis and drafted the manuscript. Githeko AK, Mukabana WR and Takken W assisted with study design and logistical issues. All authors have read and approved the final version of manuscript.

## Pre-publication history

The pre-publication history for this paper can be accessed here:

http://www.biomedcentral.com/1471-2458/12/362/prepub
